# Residual Dipolar Couplings in Structure Determination of Natural Products

**DOI:** 10.1007/s13659-018-0174-x

**Published:** 2018-06-25

**Authors:** Gao-Wei Li, Han Liu, Feng Qiu, Xiao-Juan Wang, Xin-Xiang Lei

**Affiliations:** 1 School of Pharmaceutical Sciences, South Central University for Nationalities, Wuhan, 430074 People’s Republic of China; 2 College of Chemistry and Chemical Engineering, Shangqiu Normal University, Shangqiu, 476000 People’s Republic of China

**Keywords:** NMR spectroscopy, Residual dipolar couplings, Alignment media, Structural elucidation, Natural products

## Abstract

**Abstract:**

The determination of natural products stereochemistry remains a formidable task. Residual dipolar couplings (RDCs) induced by anisotropic media are a powerful tool for determination of the stereochemistry of organic molecule in solution. This review will provide a short introduction on RDCs-based methodology for the structural elucidation of natural products. Special attention is given to the current availability of alignment media in organic solvents. The applications of RDCs for structural analysis of some examples of natural products were discussed and summarized.

**Graphical Abstract:**

This review provides a short introduction on RDCs-based methodology for the structural elucidation of natural products. Special attention is given to the current availability of alignment media in organic solvents. The applications of RDCs for structural analysis of some examples of natural products were discussed and summarized.
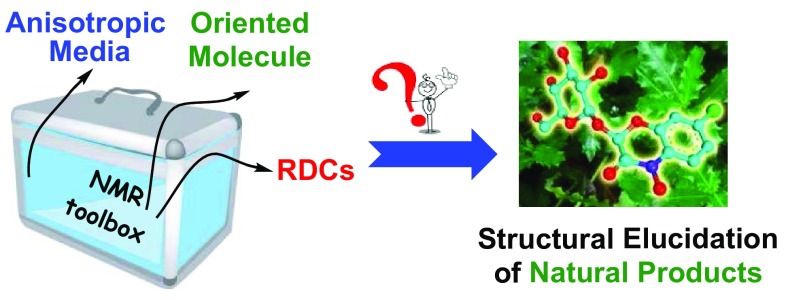

## Introduction

Solution NMR spectroscopy has been a pivotal contribution to the structural determination of natural products at the level of atomic resolution. As classical NMR parameters, chemical shifts (*δ*), scalar couplings (*J*), and nuclear Overhauser effects (NOEs) can provide valuable information about the environment of nuclear spins. However, all isotropic NMR parameters have ‘non-global’ nature: chemical shifts are typically affected by the first and second sphere of atoms surrounding the observed nucleus and usually provide only qualitative measurements. *J*-Couplings are limited to dihedral angles via three covalent bonds, and NOEs through space connectivity can merely be found up to 5 Å in favorable cases of small molecules. As soon as the chain of short-range information is interrupted by NMR-inactive nuclei, distant parts of a molecule cannot be correlated, thereby hampering the structure determination procedure. Therefore, the relative configuration of some complex structures cannot be addressed completely. In this context, residual dipolar couplings (RDCs) can provide a valuable complement for the structural elucidation of natural product, and allows orientation of angular information relative to an external reference, making it generally possible to acquire structural information from distant parts of a molecule, which could reflect the whole shape of natural products [1, 2].

The progress of RDCs in small organic molecules has been reviewed periodically since the potential application of RDCs spread to the conformation, constitution, configuration of organic molecules [3–5]. Those reviews elaborated in detail the basic principle of the theory and the entire procedures of acquisition and analysis, including pulse sequences, alignment media, calculation software. Here, we will provide a brief overview of RDCs methods and advances of alignment media and analysis methods. We discuss and outline some examples of the applications delivered by RDCs for the elucidation of natural products.

## Residual Dipolar Couplings (RDCs)

RDCs are sensitive probes for molecular structures and dynamics in solution. They provide explicit and exact descriptions of the relative bond orientations, which are highly complementary to the traditional distance restraints evidenced from NOEs. RDCs contain very valuable information for determining three-dimensional molecular structures. Dipolar couplings arise when molecular systems containing proximate pairs of magnetic nuclei are partially ordered in magnetic fields. The direct magnetic interaction between a pair of nuclear magnetic moments gives rise to the dipole–dipole interaction (e.g., ^1^H–^13^C, ^1^H–^15^N, ^1^H–^1^H). The dipolar interaction causes splitting in the signal of each nucleus involved. The resultant dipolar couplings in the solid state are on the order of kHz, leading to broad lines devoid of chemical shift resolution. The size of the resultant coupling depends on the distance between the two nuclei as well as the direction of their internuclear vector relative to the external magnetic field (*B*_0_). In isotropic solutions, the angle-dependent dipolar interactions typically average to zero, owing to the uniform distribution of all orientations (rotational Brownian diffusion). Although dipolar couplings still contribute to relaxation processes like the NOEs, a large amount of the potential structural information is lost by such an averaging. To measure the desired additional structural information without significant loss in chemical shift resolution, an intermediate state between solid and liquid must be reached, termed “alignment media.” Solute molecules are only oriented for a time average of 0.05% which reduces a 23 kHz dipolar coupling between a carbon and its directly attached proton to a RDC of only 11.5 Hz. In this way, dipolar interactions can be measured with relatively high accuracy and reasonable RDC-based line broadening. The only drawback of the method is the partial averaging due to the tumbling of the molecule and its inherent flexibility [6].

**Fig. 1:**
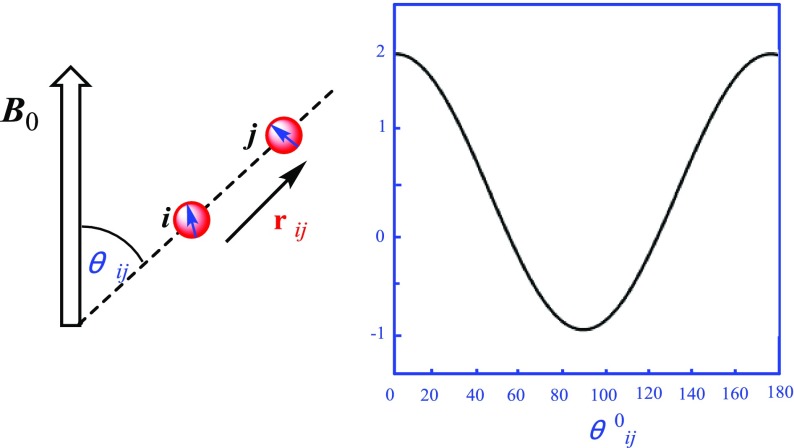
Schematic representation of two nuclear spins connected by the internuclear vector r_*ij*_ that encloses an angle *θ*_*ij*_ with respect to the external magnetic field *B*_0_; The exact formula for the dipolar coupling *D*_*ij*_ is Eq. (1)

The size of RDC for a given degree of orientation depends on the gyromagnetic ratios *γ* of the involved nuclear spins *i* and *j*, the internuclear distance r_*ij*_, and the angle *θ*_*ij*_ between the internuclear vector r and the direction of the external magnetic field *B*_0_. Their distance r_*ij*_, and the angle *θ*_*ij*_ of the internuclear vector with *B*_0_, averaged over time as indicated by the brackets, where *μ*_0_ is the vacuum permeability. The angular brackets in Eq. (1) indicate an averaging over time leading to scaled dipolar couplings (typically in the order of tenths of kHz) due to orientational reorientation compared to the static case with a maximum interaction at *θ*_*ij*_ = 0 or 180° (Fig. 1). In an isotropic solution, all orientations will be equally distributed, resulting in 〈cos^2^*θ*〉 = 1/3, a null dipolar splitting. Thus, partial alignment must be realized to produce measurable values. A different situation occurs for a solute weakly oriented in an alignment medium, as can occur in partially anisotropic media, in which the dissolved molecules are still mobile but show some slightly preferred orientations. The slightly anisotropic tumbling of the molecules is described by an alignment tensor, which is used to analyze the RDCs. Preferably, the dipolar couplings are reduced by about three orders of magnitude from several thousand hertz in the static case to only a few hertz in the partially aligned sample. In the resultant NMR spectroscopy, the RDCs add to the corresponding scalar couplings and cause a splitting *T*_*ij*_ = *J*_*ij*_ +  *D*_*ij*_, the formula for the dipolar coupling *D*_*ij*_ between two spins in a defined orientation is reflected in Eq. (1) [7].

1 $$D_{ij} = - \frac{{h\gamma_{i} \gamma_{j} \mu_{0} }}{{16\pi^{2} }}\left\langle {\frac{1}{{r_{ij}^{3} }}(3\cos^{2} \theta - 1)} \right\rangle$$

## Alignment Media for Acquiring RDCs

Until recently, the applications of RDCs are limited by the lack of the appropriate alignment media to generate a reasonable magnitude of the signals for dipolar couplings. The first spectrum reported for a partially aligned organic molecule was for benzene in a nematic liquid crystalline phase, originally reported by Saupe and Englert [8]. However, the crucial factors affecting the utilization of the “weak alignment” method for organic structure determination were the choice of the best alignment conditions, the polarity of the solvent, and the degree of order induced by the alignment media. In short, some alignment media were designed to achieve maximum dipolar couplings in anisotropic media to obtain as much structural information as possible, and other developed alignment media were optimized to allow very weak alignment without being compromised by the lower limit of the alignment strength.

**Fig. 2:**
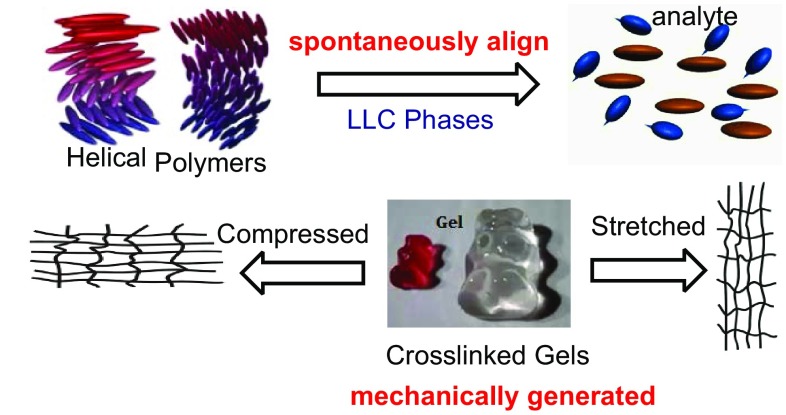
The diagram of two alignment media for RDCs analysis

Currently, there are two kinds of partially orienting media that have been usually described for studies with RDCs in anisotropic environment: alignment with lyotropic liquid crystalline (LLC), and stretched/compressed polymer gel [9–11]. However, they have different inherent mechanism. The large magnetic susceptibility anisotropy of LLC phases causes them to spontaneously align in the presence of an intense external magnetic field, and this alignment is then partially transmitted to the solvent and the molecules in solution. Correspondingly, the anisotropy was mechanically generated either by compressing or stretching the gels, and the degree of alignment is tunable. The two methods for aligning molecules have been sketched out in Fig. 2.

### Lyotropic Liquid Crystalline

LLC phases have been observed in many biomolecules (e.g. lipids, cellulose, and DNA), and these form characteristic mesophases, a state of matter that combines the long-range order of crystals with the mobility of an isotropic liquid. In recent years, the helical lyotropic systems such as amino acid-based peptide phases, chiral high polymers, carbon-based graphene oxide (GO) sheets, and other liquid crystals have been introduced for measuring RDCs (Table 1). Notably, some of cholesteric phases generally function within relatively narrow ranges of temperature and solvent applicability. They have also excellent enantiodiscrimination capabilities with most apolar organic solvents like CDCl_3_, CD_2_Cl_2_, or THF. The cholesteric phases can also be used with the polar organic solvent CD_3_OD, DMF and in mixtures with up to DMSO.

**Table 1:** The different LLC media according to structure types of polymers

Types of polymers	LLC	Compatible solvents	Chirality	References
Polypeptides	PBLG/PBDG	CDCl_3_, CD_2_Cl_2_, DMF, THF, Dioxane	Chiral	[26]
	PCBLL/PCBDL	CDCl_3_	Chiral	[27]
	PELG/PEDG	CDCl_3_, CD_2_Cl_2_	Chiral	[28]
	PSMBLG/PSMBDG	CDCl_3_	Chiral	[29]
	PPLA/PPDA	CDCl_3_,TCE-*d*_2_	Chiral	[30]
	ACHC-rich β-peptides	D_2_O	Chiral	[31]
	AAKLVFF	CD_3_OD, CD_3_OD/acetone, CD_3_OD/DMSO	Chiral	[22]
Polyguanidines	(*R*)-PPEMG	CDCl_3_	Chiral	[15]
Polyarylacetylenes (PPA)	L-Ala-based PPA	CDCl_3_	Chiral	[16]
	L-Phe-based PPA	CDCl_3_	Chiral	[17]
	L-Val-based PPA	CDCl_3_		[18]
Polyarylisocyanides (PPI)	L-Ala-based PPI	CDCl_3_, CD_2_Cl_2_, THF	Chiral	[19]
Polyisocyanopeptides (PIP)	l,l-PIAF-OBn	CDCl_3_		[21]
Graphene oxide	GO	D_2_O-DMSO, D_2_O-CD_3_CN, D_2_O-Acetone	Achiral	[23]
	GO-*g*-TFEMA	DMSO	Achiral	[22]
Supramolecules	SaS-LLCs	CDCl_3_,CDCl_3_-CCl_4_, THF	Chiral	[32]
Others	DSCG	D_2_O	Achiral	[25]
	C_12_E_5_	D_2_O	Achiral	[33]
	C_12_MPB	D_2_O	Achiral	[34]

The NMR system in weakly organic aligning media was pioneered by Lesot et al. for enantiodiscrimination purposes and determination of the RDCs [12]. Known examples include poly-*γ*-benzyl-l/d-glutamate (PBLG/PBDG), poly-*ɛ*-carbobenzyloxy-l/d-lysine (PCBLL/PCBDL), and poly-*γ*-ethyl-l/d-glutamate (PELG/PEDG). RDCs were successfully used to determine the structure of small molecules, such as distinguishing between the two diastereotopic protons on C-20 of strychnine. Unexpectedly, these synthetic polypeptides showed an amazing chiral discrimination power when used as the chiral selector, as well as a large ability to dissolve organic molecules [13, 14].

Since rigid-backbone helically chiral polymers can form anisotropic phases at rather low concentrations in a variety of non-polar solvents, they are interesting candidates for the development of new chiral alignment media. Reggelin et al. have developed a chiral poly-(*N*-methyl-*N*′-((*R*)-1-phenylethyl) guanidine ((*R*)-PPEMG), enantiodiscriminating orienting media for isopinocampheol based on helically chiral polyguanidines. The (*R*)-PPEMG exhibited a cholesteric liquid crystalline phase at a critical concentration of 18.7% in chloroform [15]. Furthermore, the alanine, valine and phenylalanine-derived polyarylacetylenes have been used to demonstrate the differentiation of enantiomers of strychnine and isopinocampheol by Reggelin’s and Berger’s groups [16–18]. These polymers were found to be excellent alignment media for the RDC measurements in CDCl_3_ because they combine narrow line widths from the analyte and optimum alignment tensors for the measurement of RDCs with pronounced enantiodifferentiating capabilities. This chiral polyarylisocyanide bearing an l-Ala residue also exhibited liquid crystalline phases in THF and CD_2_Cl_2_ solutions at a concentration of 17.9% with dozens of Hz quadrupolar splittings of the solvent signal and showed a smaller degree of alignment compared to PBLG or (*R*)-PPEMG [19]. Note that the freshly prepared Ala-based polyarylisocyanide (PAI) is in a kinetically controlled conformation which is usually not the one with the maximum helicity. Therefore, it is necessary to anneal the Ala-based PAI for some time in toluene to reach the thermodynamically stable conformation [20].

In recent years, our group has paid continuing attention to the development of novel alignment media for acquiring RDCs. We have developed the l,l-PIAF-OBn as a novel and effective alignment medium in CDCl_3_, and they have been successfully used for the RDCs measurement of strychnine and triptolide. This work represented the first example of a polymeric LLC which was stable at low critical concentration. Importantly, the excellent solubility and intrinsic low viscosity allowed us to acquire good quality NMR spectra with narrow lines [21]. The self-assembled oligopeptide has the sequence of AAKLVFF, which was derived from a fragment of the amyloid β-peptide, afterward, we have successfully developed this liquid crystal-forming AAKLVFF peptide as a versatile alignment medium. This work was also the first example of a MeOH-based lyotropic liquid crystalline as an aligning medium at very low concentration, and seven natural products containing very different functional groups were measured to demonstrate the wide compatibility and applicability for the RDC analysis of natural products [22].

**Fig. 3:**
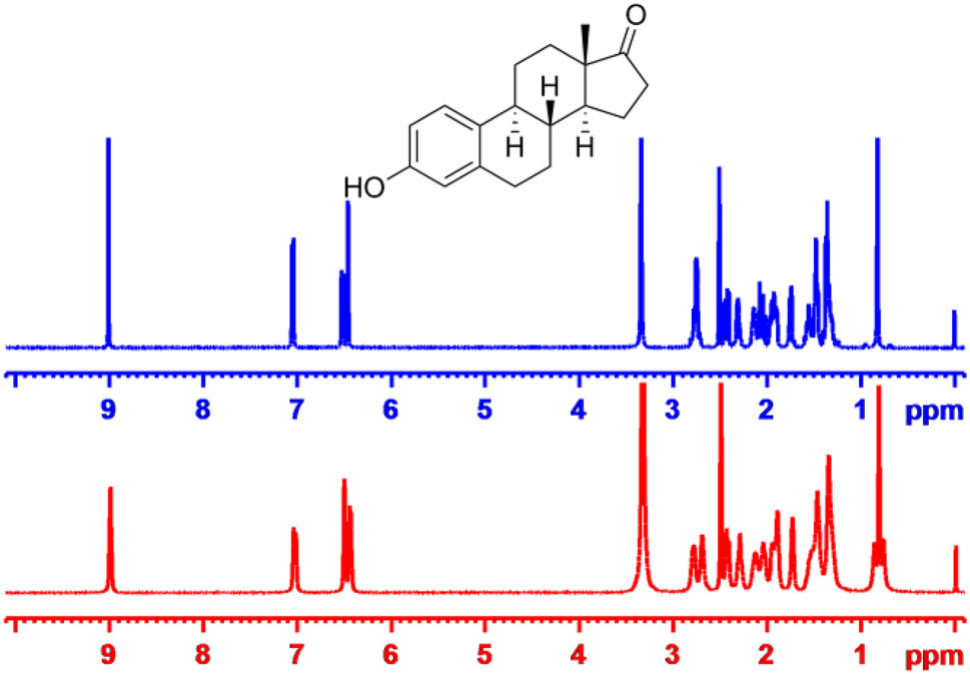
^1^H NMR of estrone in the isotropic (blue) and anisotropic GO-*g*-TFEMA (red)

In another our previous work, we have developed the graphene oxide-based (GO) LLCs which was a novel anisotropic orientation medium whose low critical concentration, and facile scalability comply with the criteria for an ideal orientation medium. The GO-based LCs can produce anisotropic interaction to align small molecule and showed unexpected advantages, including the unprecedented characteristic of high-quality NMR spectra without any background signals as a result of the rigidity and high molecular weight of GO molecules. The media was compatible with a broad range of solvents (except pure DMSO) [23]. To enhance dispersibility of GO nanosheets in conventional pure organic solvents, we decided to further optimize the GO-based LC phases by grafting polymer brushes to get an alignment medium that would be soluble in pure DMSO. To our great delight, the GO-*g*-TFEMA showed excellent solubility and dispersibility, and exhibited very low viscosity in pure DMSO [24]. The imperfect features of our previously reported unmodified GO LCs was the strong π-π interactions that occurred between GO sheets and analytes, In the new GO-*g*-TFEMA phase, potential π-π interactions are expected to be blocked by steric hindrance arising from the alkyl chain brushes grafted onto grapheme oxide. A high quality proton NMR spectrum was obtained under anisotropic conditions (Fig. 3), suggesting that the aggregation of the GO layer and analytes could indeed be successfully prevented by the GO grafting polymer chains.

Disodium cromoglycate (DSCG) is noteworthy because its chromonic phases are oriented in external magnetic fields, allowing the observation of anisotropic NMR observables such as quadrupolar splittings or RDCs. Armando et al. explored the scope of DSCG as a weakly aligning phase for practical applications in structural elucidation of organic molecules. The medium was compatible with a variety of samples of different polarity, and the degree of alignment could be tuned by varying the mesogen concentration, temperature, and the addition of brine [25].

Table 1 lists the different types of LLC media for RDC measurements.

### Gel-based Alignment Media

The incorporation of analytes into an anisotropically stretched/compressed polymer gel to orient the molecules was another important strategy. A common preparation scheme involved placing a dry and cross-linked polymer stick into an NMR tube. The polymer was then swollen by the addition of a solvent. The swelling gel reached the glass walls and any further swelling would automatically result in stretching. Solute molecules diffused into the stretched gel were partially oriented by the anisotropic gel matrix. The shortcoming was that it typically takes several days for the solute molecule to diffuse into the polymer gels.

The first gel-based alignment medium suitable for apolar solvents was cross-linked polystyrene (PS), which was compatible with all the apolar solvents (CDCl_3_, CD_2_Cl_2_, C_6_D_6_, THF, and dioxane) [35]. This gel was very robust and has been applied to several systems. In a perdeuterated version, the gel matrix was of high interest for solute molecules. The almost signal free and scalable alignment medium enabled the RDC measurement at very low concentrations and allowed the acquisition of any kind of homonuclear correlation experiments [36]. Poly(vinyl acetate) (PVAc) is another robust polymer matrix applicable for more polar solvents like methanol, acetone and acetonitrile, and worked reasonably well with DMSO and DMF [37]. Moreover, the cross-linked poly(dimethylsiloxane) (PDMS) also formed a superb alignment medium for molecules in weakly polar organic solvents generated by irradiation with β-rays or chemical synthesis [38, 39]. This extended the solvent range to more apolar solvents like hexane in a superb alignment medium.

Poly(methyl methacrylate) (PMMA) gels prepared by copolymerizing methyl methacrylate and various amounts of ethylene glycol dimethacrylate. Cross-linked PMMA had very good aligning properties for small molecules in chloroform. The alignment was scalable and depended on the cross-link density, similar to other gels [40]. Cross-linked PEO can be produced from linear PEO either by irradiation (*γ*-PEO) or by chemical modification of the terminal hydroxyl group, or by a combination of both protocols [41]. The solvent compatibility of both *γ*-PEO and PEOMMA was outstanding. With the exception of hexane, considerable swelling was observed with all test solvents including nonpolar solvents (like benzene, toluene, and dioxane) and polar solvents such as MeOH, DMSO, and water. Such a wide compatibility of PEO came with only a few restrictions. Gelatin in the form of *gummibärchen* was applied for the initial proof of principle that alignment was possible. Luy et al. demonstrated that as chiral alignment media, these stretched gelatin gels provided not only the structural information through RDCs, but also a way for discriminating enantiomers for the measurement of enantiomeric excess [42].

Table 2 lists the stretched/compressed gels and solvents used for RDC measurements.

**Table 2:** Cross-linked polymer gels and their solvent compatibilities and properties

Gels	Compatible solvents	Chirality	References
Gelatin	D_2_O	Chiral	[42]
e^−^-Gelatin	D_2_O, DMSO	Chiral	[43]
Collagen	D_2_O	Chiral	[44]
PAA	D_2_O	Achiral	[45]
PH	D_2_O, DMSO, DMF	Achiral	[46]
APhES-PH	D_2_O, DMSO, CD_3_OD	Chiral	[47]
PAN, dPAN	DMSO, DMF	Achiral	[48]
PEO	D_2_O, CD_3_CN, CD_3_OD, DMSO, DMAC, acetone, THF, CDCl_3_, CD_2_Cl_2_, C_6_D_6_, dioxane, *n*-hexane	Achiral	[41]
PVAc	CDCl_3_, CD_2_Cl_2_, C_6_D_6_, CD_3_OD, CD_3_CN, DMSO, DMF, acetone, EtOAc, dioxane	Achiral	[37]
PMMA	CDCl_3_, CD_2_Cl_2_, C_6_D_6_, CD_3_CN, acetone, EtOAc	Achiral	[40]
PS, dPS	CDCl_3_, CD_2_Cl_2_, THF, C_6_D_6_, dioxane	Achiral	[35]
PDMS	CDCl_3_, CD_2_Cl_2_, THF, C_6_D_6_, dioxane, *n*-hexane	Achiral	[38]
PBLG gel	CDCl_3_, CD_2_Cl_2_, THF, C_6_D_6_, Dioxane	Chiral	[49]
p-HEMA	DMSO	Achiral	[50]
p-DEGMEMA	CD_3_OD	Achiral	[51]

## RDCs Extraction and Data Analysis

**Fig. 4:**
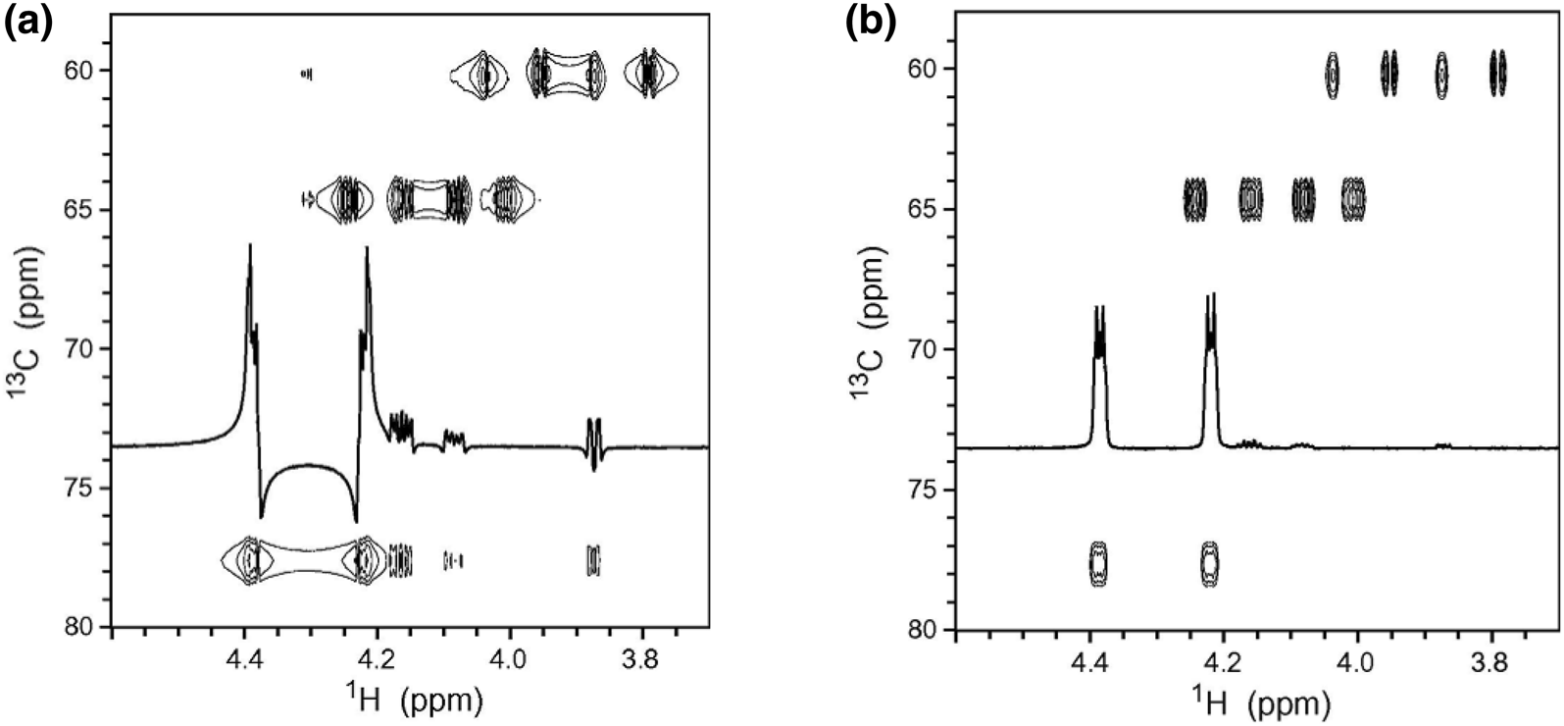
Spectral region of conventional F_2_-coupled HSQC (**a**) and the CLIP-HSQC spectrum (**b**) of strychnine in CDCl_3_ for a mismatched delay for heteronuclear coherence transfer

RDCs were generally acquired for C–H, C–C, N–H and H–H vectors. In principle, most experiments for measuring scalar couplings can be used to extract dipolar couplings, like conventional experiments such as heteronuclear multiple-quantum correlation (HMQC) or heteronuclear single-quantum correlation (HSQC) [52]. The most readily obtainable coupling was the one-bond ^1^*D*_C–H_, which can be measured in a F_2_-coupled HSQC spectrum as the ^1^*J* + ^1^*D* splitting of the ^1^H resonance coupled to ^13^C [53]. The ^1^*D*_C–H_ coupling was afforded by subtracting the ^1^*J* splitting measured in the same spectrum for a non-aligned sample. The conventionally coupled HSQC spectrum has strong dispersive antiphase components and contributions from long-range connectivities which are absent through the clean inphase (CLIP) pulse sequence (Fig. 4). Moreover, an ideal strategy has been developed to circumvent individual phasing of the multiplet components by the clean antiphase (CLAP) pulse sequence that can remove dispersive antiphase components prior to detection [54].

Recently developed and fully homo-decoupled HSQC spectra have been recorded through a new pulse sequence element, “perfectBIRD”, which can collapse splittings due to geminal couplings between diastereotopic methylene protons. The *J*-scaled BIRD HSQC spectra of acceptable quality can be obtained to allow highly precise measurements of one-bond RDCs in the high-resolution proton dimension, even in weakly aligned media [55].

**Fig. 5:**
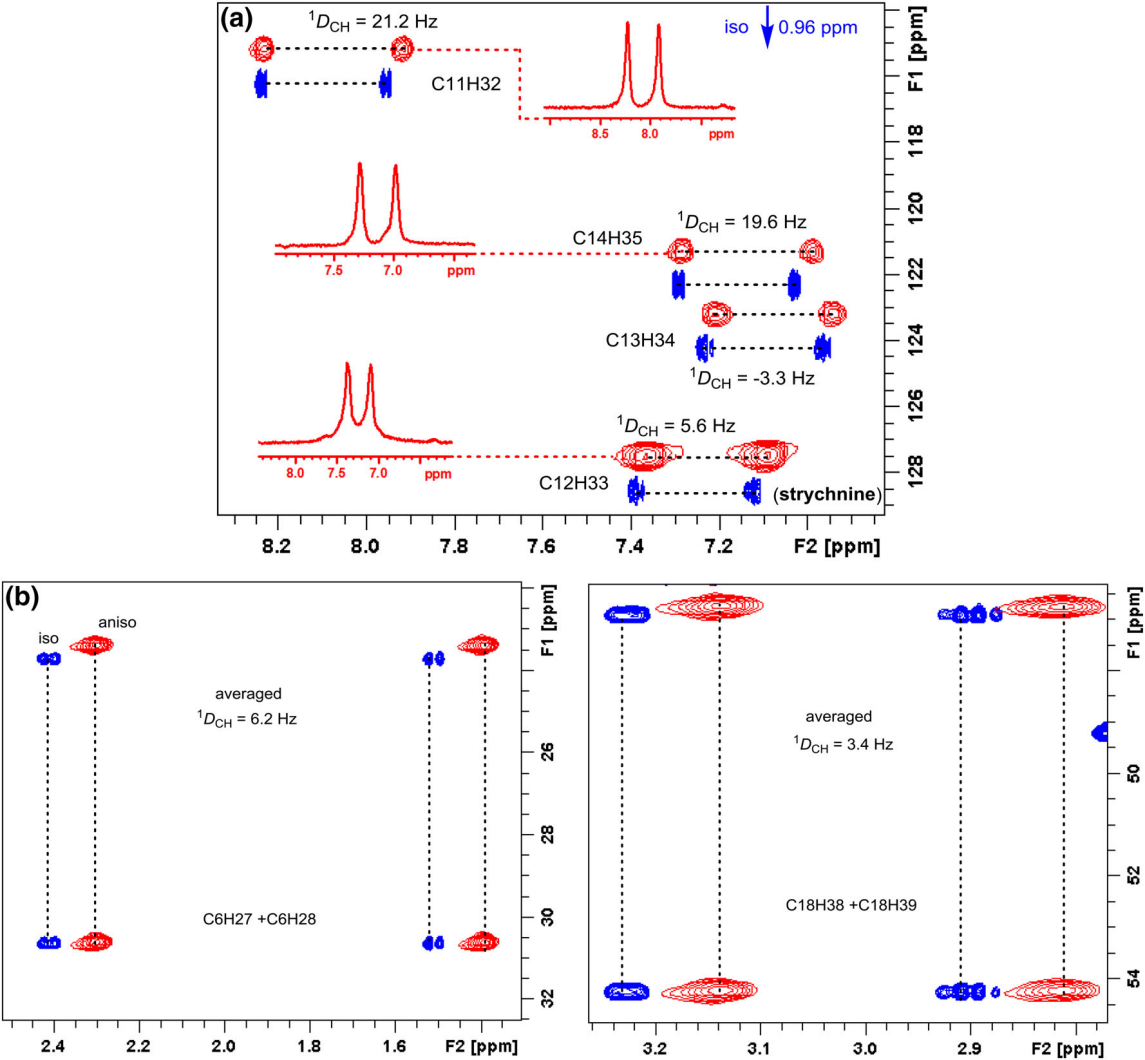
(**a**) Expanded CLIP-HSQC spectra of strychnine in the isotropic CDCl_3_ phase (blue contours) and in anisotropic l,l-PIAF-OBn LCs (red contours). The inserted trace from the anisotropic 2D spectra (C11/H32, C12/H33, C13/H34 and C14/H35) illustrates the favorable line widths. (**b**) Expanded regions of methylene C6/H27 and C6/H28, C18/H38 and C18/H39 signals in the JSB-HSQC experiment for measuring the ^1^*D*_CH_ couplings

This then requires the acquisition of two sets of spectra: one in isotropic conditions, and the second in anisotropic media, One-bond proton-carbon *J*-couplings (^1^*J*_CH_) in the isotropic sample and the sum of the one-bond dipolar and *J*-couplings (so called total coupling ^1^*T*_CH_) in the presence of the anisotropic phase were collected using appropriate NMR pulses experiment. RDC values (^1^*D*_CH_) were extracted from the obtained spectra using the relation ^1^*D*_CH_ = ^1^*T*_CH_-^1^*J*_CH_. In a recent study, we successfully acquired RDCs of the highly rigid strychnine using F_1_-coupled and F_2_-coupled experiment, respectively. Figure 5 shows RDCs calculation data of F_2_-coupled CLIP-HSQC and F_1_-coupled *J*-scaled BIRD HSQC spectra collected in isotropic and anisotropic media.

The measurement of ^n^*D*_H–H_ and ^n^*D*_C–C_ is another useful type of dipolar coupling for natural product stereochemistry analysis. The direct extraction of homonuclear proton-proton RDCs in the aligned sample has been difficult because of the multitude of long-range RDCs contributing to the proton multiplets. For proton signals without much overlap, a selective one-dimensional directed COSY experiment was feasible for measurement of ^n^*D*_H–H_ [56]. For unresolved proton multiplets, a 2-D constant time COSY experiment (CT-COSY) was proposed for the quantitative extraction of the RDCs [57]. For fully labeled ^13^C samples, sign-sensitive measurement of RDCs can be achieved using exclusive correlation spectroscopy (E. COSY) when multiplets were not too strongly broadened or by using 2D ^13^C-^13^C constant time COSY experiments [58]. The INADEQUATE spectrum has also been shown to provide RDCs along with ^1^*D*_C–C_ and even ^2^*D*_C–C_ couplings [59]. Such carbon–carbon RDCs are one order of magnitude smaller than the corresponding proton-carbon or proton-proton RDCs, which must be taken into account. The same problem occurs for long range proton-carbon RDCs, which are very difficult to measure with the necessary accuracy.

Griesinger and coworkers found that organic molecules in orienting media were amenable to the measurement of long-range heteronuclear couplings [60]. An approach based on the HMBC pulse sequence has been developed to achieve the necessary precision to measure ^2,3^*D*_C–H_ values for menthol dissolved in PBLG and chloroform. The experimental RDCs matched quite well with the values calculated from the X-ray structure.

**Fig. 6:**
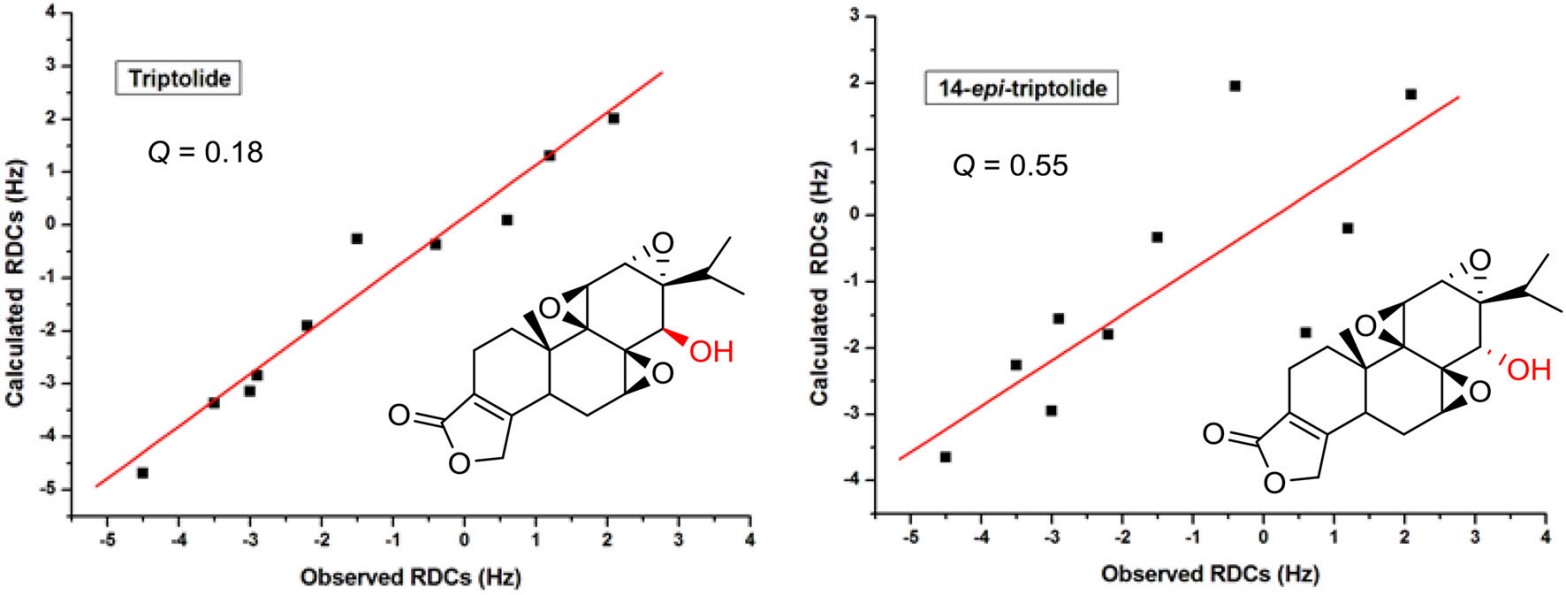
Correlation between the experimental ^1^*D*_CH_ and the back-calculated RDC of triptolide in l,l-PIAF-OBn LCs using the X-ray structure of triptolide/14-*epi*-triptolide as the input. The RDC fitting was performed with SVD using MSpin software package

Analysis of RDCs in terms of Saupe order matrices provided a concise description of both orientation and motional properties. To verify the structure of solute using the acquired RDCs, we need at least five independent RDCs and calculate the alignment tensor using the singular value decomposition (SVD) methodology [61] with the program package MSpin [62]. Theoretically predicted RDCs were calculated from the computed alignment tensor by using the DFT-optimized structure as the input and further compared with the experimental determined ones. The SVD method will always generate an alignment tensor from the best fitting of the experimental RDCs (*D*_exp_) to the proposed structure. Using the calculated alignment tensor, the RDC value for each internuclear vector will be back calculated (*D*_calc_). SVD is the most commonly used method, but similar results are obtained if the alignment tensor is determined by minimizing the difference between the observed (*D*_exp_) and the back-calculated (*D*_calc_) RDCs as a function of the matrix elements of the tensor A using a least-squares method. The quality of the fitting (*D*_exp_ *vs. D*_calc_) is commonly expressed in terms of the Cornilescu quality factor *Q* (*Q *= rms (*D*^exp^ − *D*^calc^)/rms *D*^exp^) [63], if the RDCs are measured with good accuracy, the lowest *Q* corresponds to the correct structure. Here, a biologically active triptolide was chosen as the analyte, we examined the fit of the experimental RDC data to the two stereoisomers. SVD fitting of these data to the structures of both isomers generated by X-ray structures as the input, showed a *Q* factor of 0.18 for the triptolide isomer and of 0.55 for the 14-*epi*-triptolide isomer. This result clearly demonstrates the value of the RDC data acquired in the l,l-PIAF-OBn for the stereochemical elucidation of triptolide (Fig. 6).

## RDCs in Structural Elucidations of Natural Products

The RDC-based stereochemical assignment of natural products usually included relative configuration determination, diastereotopic assignment, and conformational analysis. A general problem with many natural products was that they were isolated in very small amounts. Alignment media, even working under optimal experimental conditions, introduced significant line broadening that leaded to a considerable loss in signal-to-noise. Hence, such experiments required a minimum amount of sample that was normally higher than the amount available. In order to overcome this problem, Griesinger et al. had developed a PH gel with a diameter that was suitable for use in a 1.7 mm MicroCryoProbe significantly reducing the requirement of sample amount and making the RDC measurement and structural analysis of rare natural products possible. The gels were polymerized in a 2 mm chamber and, after washing and rinsing, they fitted neatly in 1.7 mm NMR tubes and can be re-swollen in only milligrams of DMSO. We noted an important sharpening of the NMR lines as compared to gels prepared in 5 mm tubes, which attributed not only to better shimming of the smaller-diameter tube, but also to a higher homogeneity of the gel inside the thinner tube.

**Fig. 7:**
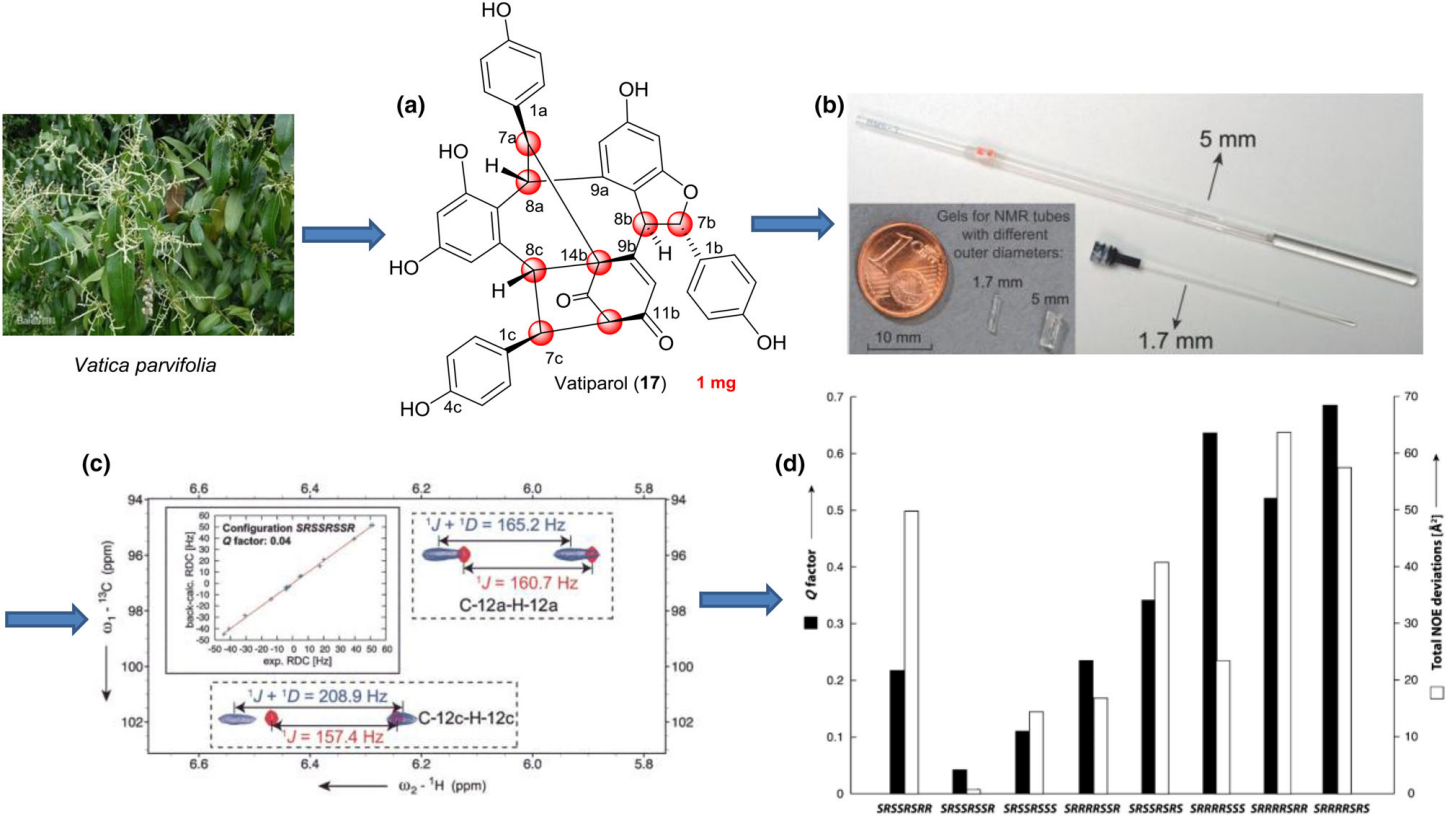
Schematic representation of the steps involved in determination of natural product stereochemistry by RDC analysis. (**a**) Structure of vatiparol (**17**). (**b**) Comparison between the gels for 1.7 and 5 mm NMR tubes. (**c**) The ^1^*D*_C–H_ values are extracted as the difference of the splitting between isotopic and anisotropic signals. (**d**) Comparison of *Q* factors of eight possible diastereomers by NOE deviations and RDC fitting

This combined NMR/chiroptic procedure has been successfully applied to the determination of the absolute configuration of several natural products. Here, the instructive example illustrated the application of RDCs and chiroptics for absolute configuration of vatiparol (**17**) in Tan’s laboratory (Fig. 7) [81]. Vatiparol, a resveratrol trimer, contained eight stereocenters, C7a, C8a, C7b, C8b, C12b, C14b, C7c and C8c, where the relative configuration was first established by NOE-based NMR analyses. The *J* coupling analysis yielded a *syn* relative configuration for C7a/C8a and an *anti* arrangement for C7b/C8b. Based on geometric considerations the relative configuration of C7a/C14b should be *anti* and C12b/C14b *syn*. Using these restraints, the 128 possible configurations of vatiparol can be reduced to 16, which were *RSRRSRSS*, *RSRRSRRS*, *RSRRSRRR*, *RSSSSRRS*, *RSRRSRSR*, *RSSSSRRR*, *RSSSSRSS* and *RSSSSRSR* for C7a, C8a, C7b, C8b, C12b, C14b, C7c and C8c, respectively, and their enantiomers. The remaining conformation/configuration combinations were optimized with DFT B3LYP/6-31G* level of theory. To evaluate the possible diastereomers they tested the optimized conformation of each configuration against the NOE data. Altogether 56 NOE distances were extracted from a NOESY spectrum, including 41 long-range NOEs defined here as NOEs between two protons separated by more than four bonds. The NOE deviations fitting showed clearly that the configuration *SRSSRSSR* has the lowest total NOE violations out of the eight possible diastereomers. Furthermore, no significant individual NOE violation can be found for this configuration.

For evaluating different diastereomers independent of the NOE analysis, 16 ^1^*D*_CH_ values were extracted for subsequent measurement in CLIP-HSQC experiments, and were analyzed from the potentially rotatable phenyl rings by using SVD fitting. Consistent with the NOE analysis, the smallest *Q* value of 0.04 was found for *SRSSRSSR* (or its enantiomer). In order to fully define the absolute configuration, the torsional information was then employed in the computation of ECD and ORD curves, since proper knowledge of the conformational preference of the phenyl chromophores was essential to accurately describe the chiroptical properties. Therefore, although RDCs alone can’t be used to determine absolute configuration, the structural information they provide was instrumental when chiroptical techniques were used for this purpose.

4,6-Diacetylhygrophorone A^12^ (**25**) (the structure shown in Fig. 9) [83], is 2-cyclopentenones with hydroxy or acetoxy substituents at C4 and/or C5, and an dodecyl alkyl chain is attached to C5, and its relative configuration of the stereogenic centers in the cyclopentenone ring unambiguously was achieved by fitting several structure proposals to the experimental data using anisotropic RDC parameters. The more challenging problem of the rotation of the side chain along the C-5–C-6 bond can be treated by calculating low-energy geometries for the (+)- and (−)-synclinal and antiperiplanar states of the OH–C5–C6–H6 torsion and using these conformer geometries as a cross-validation in the RDC fitting. A total of four one-bond ^1^*D*_CH_ and four long-range ^2/3^*D*_CH_ including their relative signs could be extracted in PBLG/CD_2_Cl_2_ medium. In each case a rigid geometry is assumed, and all eight RDCs are included in the fitting process. The 4*R*,5*S*,6*R* relative configuration fits the experimental data best, as evidenced by the lowest quality factor.

**Fig. 8:**
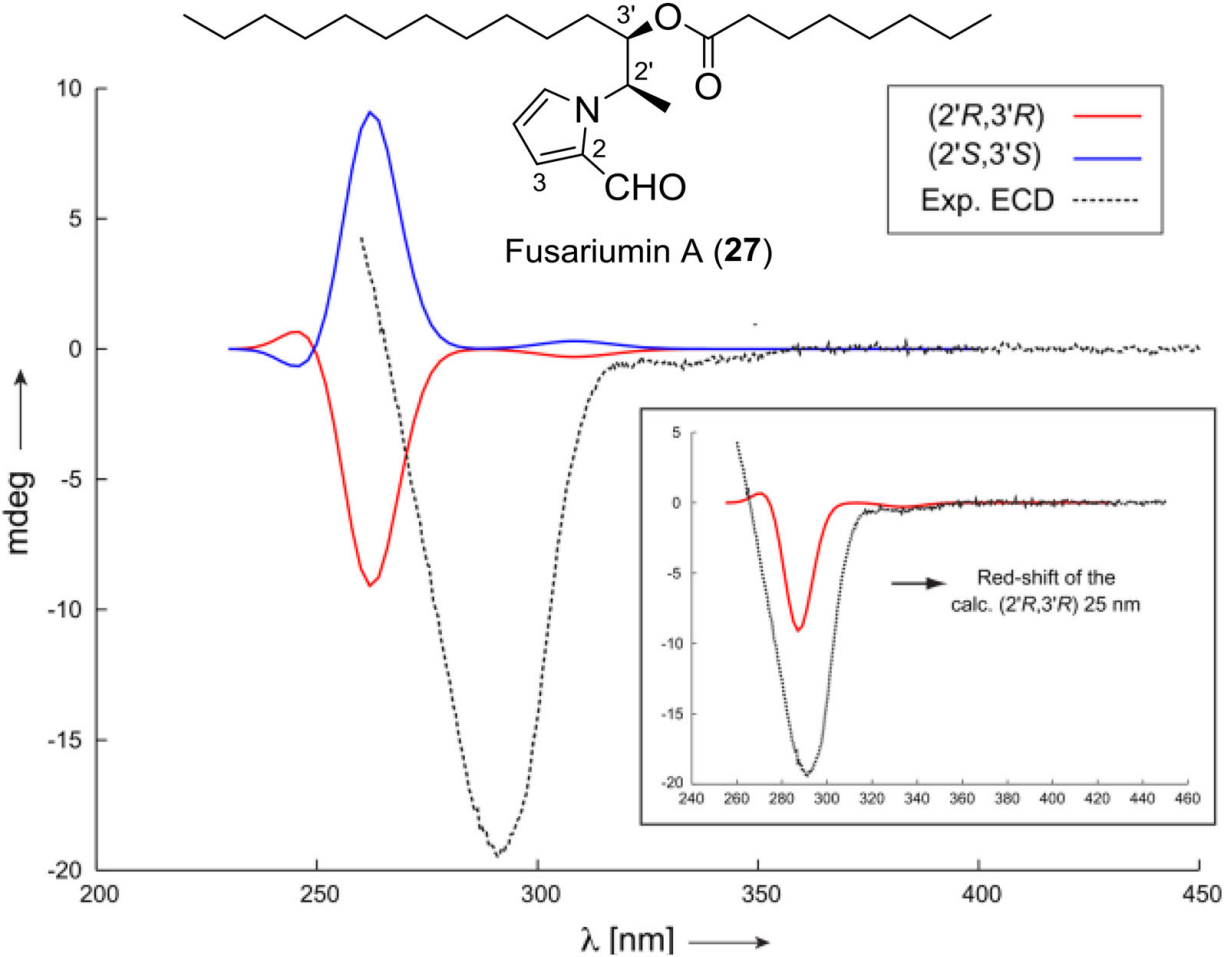
Comparison of experimentally measured ECD spectrum of fusariumin A (**27**) to the calculated ones using the RDC determined conformer (both enantiomers)

Another example of the interplay between RDCs and chiroptical spectroscopies is the determination of the absolute configuration of alkylpyrrole derivative fusariumin A (**27**) by Liu and coworkers in 2016 [89]. The molecule that contains a large number of potentially rotatable bonds, leading to a complex conformational space that is difficult to sample, the two neighboring stereocenters C2′ and C3′ using ^2,3^*J*-couplings exclusively had failed, and only limited amount of sample (about 1 mg) was available. So, the slim PH-gel was used to acquire the RDC data again. 10 ^1^*D*_CH_ were extracted from the spectra and they were used to calculate the alignment tensor for each possible conformation using the SVD method. By Comparison of the *Q* factors of the RDC fitting and Newman projections of the conformers. Additionally, NOE peaks that are relevant in the configurational and conformational analysis have been integrated, the results clearly show that the two lowest *Q* factors were obtained for relative configuration (2′*R*,3′*R*) or its enantiomer. Furthermore, the preferential population of a single local conformation around the dihedrals of C3′–C4′, C2′–C3′ and C2′–N was determined by the excellent fit of the RDC data, and was further corroborated by the *J*-coupling and NOE analysis. The long alkyl side chains were not analysed further, since they are assumed to be predominantly all-trans and were shown to have little influence on the RDC enhanced NMR analysis presented here. To determine the absolute configuration, ECD spectra of both enantiomeric forms have been calculated with time-dependent density functional theory (TD-DFT). Based on the ECD data, the absolute configuration of fusariumin A (**27**) was assigned as (2′*R*,3′*R*) (Fig. 8).

**Fig. 9:**
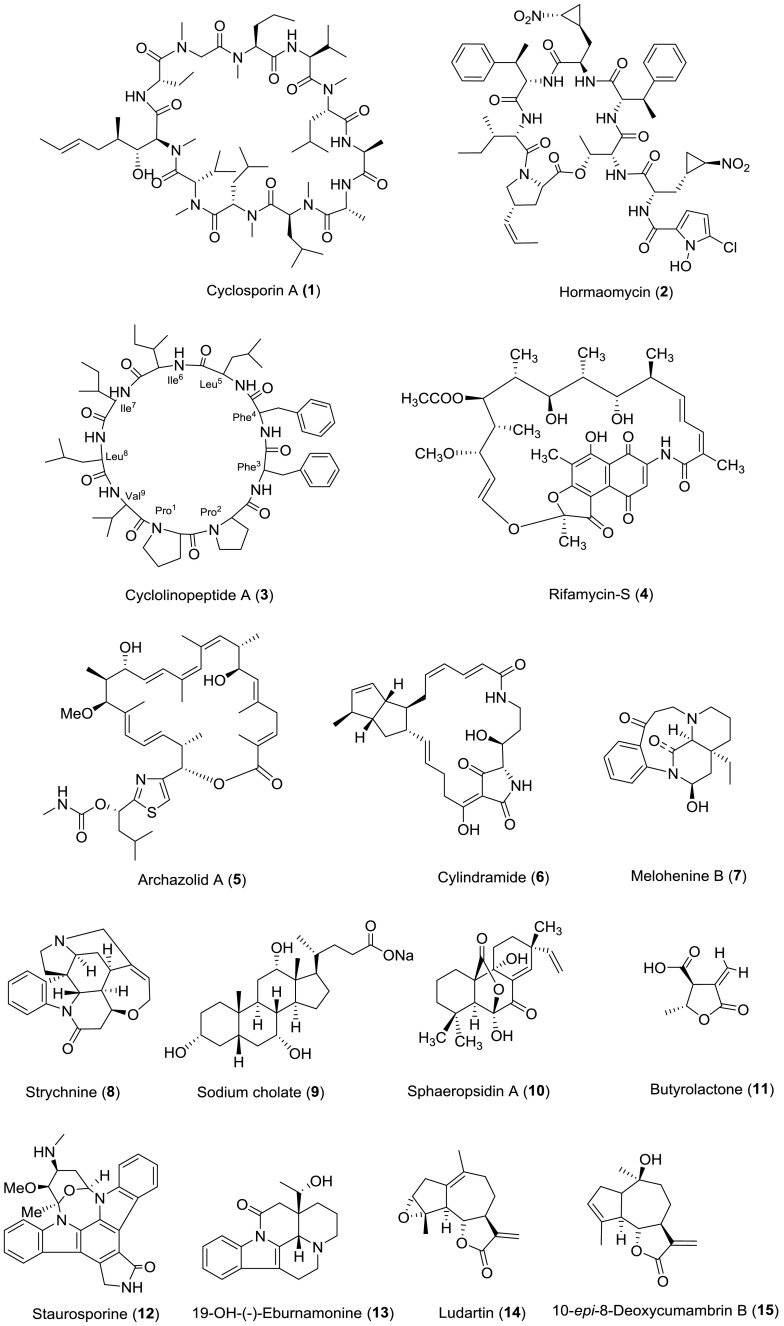

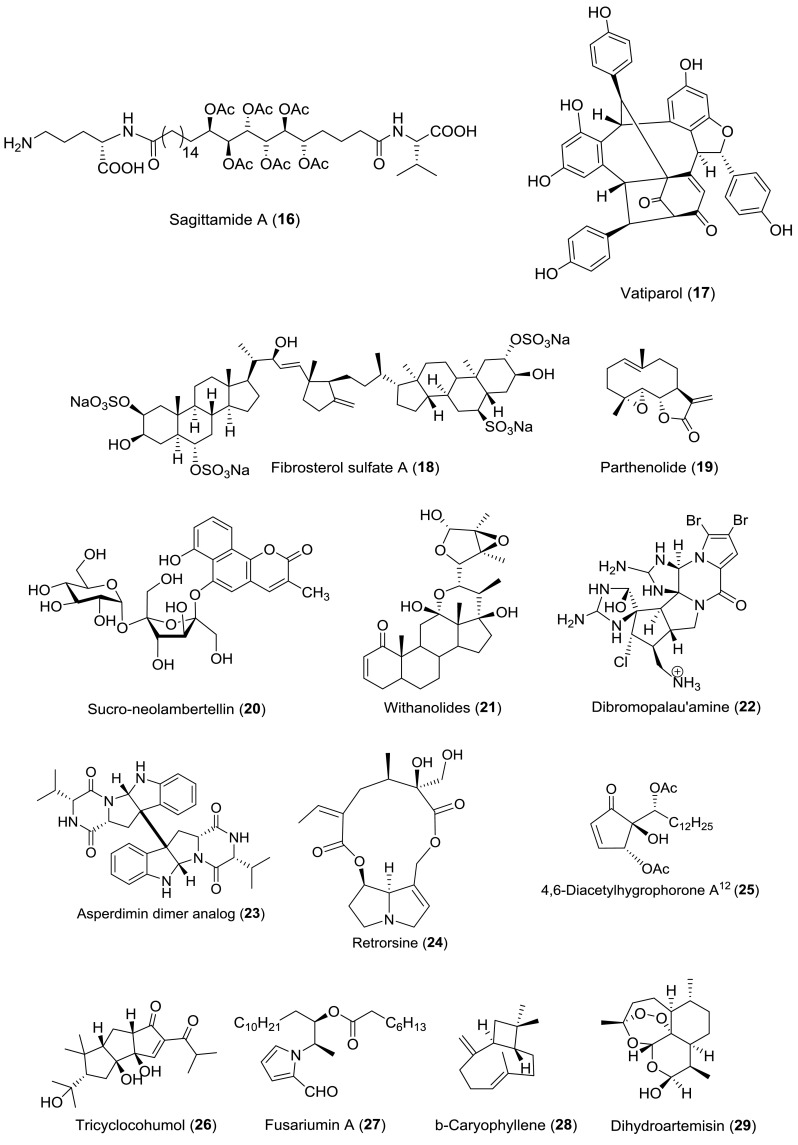

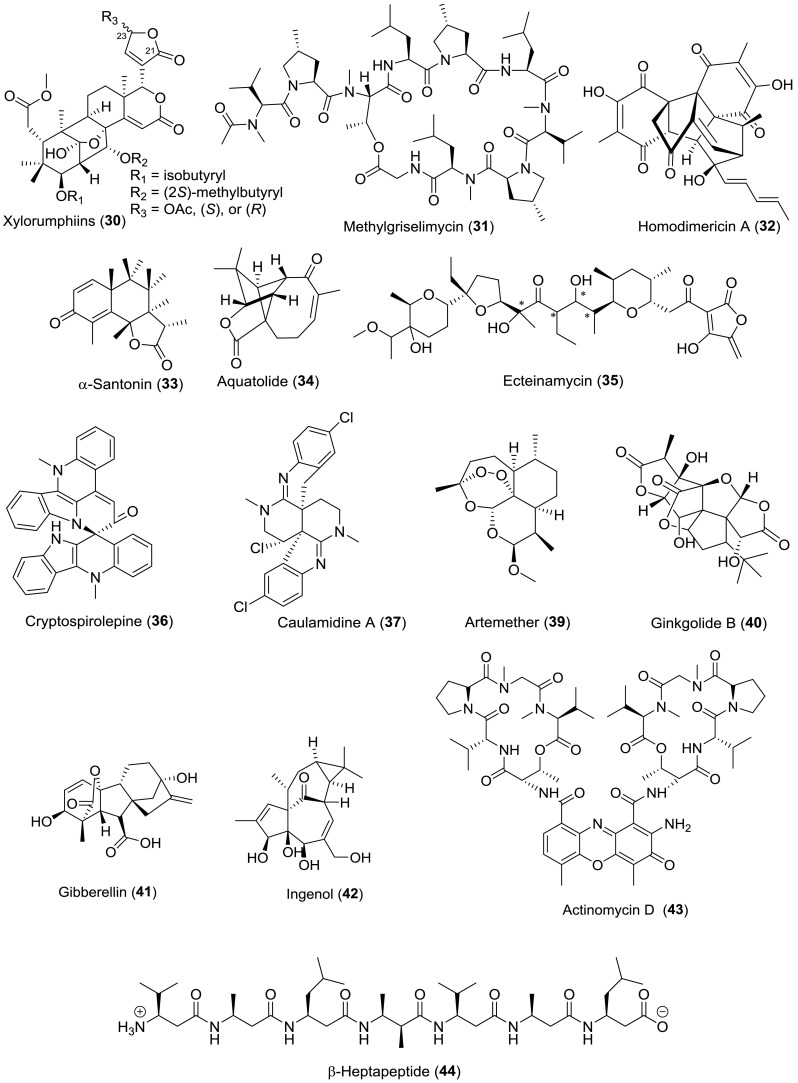
Natural product with the stereochemistry assigned by RDC measurements

The above-mentioned three cases represent some examples for the application of RDCs at very low concentrations to stereochemistry determination. RDCs are nowadays an excellent tool for the configurational/conformational analysis of natural products. Table 3 lists all the natural products (all structures shown in Fig. 9) with stereochemistry addressed via RDC measurements over the past 15 years (2003–2018), and for more examples are not illustrated in detail for structural determination, the graphical overview of the workflow of RDC analysis is given in Fig. 9.

**Table 3:** Natural products stereochemistry applications of RDCs

Natural products	Source organism	Alignment medium	Year of assignment	Direction of application	References
Strychnine	*Strychnos*	PBLG/CDCl_3_, PELG/CDCl_3_, PS/CDCl_3_	2003–2005	Prochiral assignment	[14, 26, 64]
Sodium cholate	*Bos taurus domesticus Gmelin*	PAA/D_2_O	2003	Relative configuration	[65]
Cyclosporin A	*Trichoderma polysporum*	PDMS/CDCl_3_	2005	Conformation	[66]
Sphaeropsidin A	*Diplodia cupressi*	PVAc/DMSO	2005	Prochiral assignment	[37]
*γ*-Buty rolactone	*Asteraceae, Magnoliaceae, Winteraceae, Lauraceae*	(C_12_E_5_)/*n*-hexanol/D_2_O	2006, 2009	Relative configuration	[67, 68]
Hormaomycin	*Streptomyces griseoflavus*	PH/DMSO	2006	Conformation	[69]
Sagittamide A	*Didemnid ascidian*	PH-PDMAA, PAN/DMSO	2007	Relative configuration	[70]
Ludartin	*Steυia yaconensisνar. subeglandulosa*	PMMA/CDCl_3_	2008	Relative configuration	[40]
Staurosporine	*Streptomyces staurosporeus*	dPS/CDCl_3_	2008	Relative configuration,	[36]
Cylindramide	*Halichondria cylindrata*	PAN/DMSO	2008	Conformation	[71]
Archazolide A	*Archangium gephyra*	PH-PDMAA/DMSO	2008	Relative configuration, conformation	[72]
Sucro-neolambertellin	*Lambertella* sp.	PH-PDMAA, PAN/DMSO	2008	Relative configuration	[73]
Withanolides	*Jaborosa parviflora*	PMMA/CDCl_3_	2009	Absolute configuration	[74]
Tricyclocohumol	*Humulus lupulus* L.	dPAN/DMSO	2009	Relative configuration	[75]
10-*epi*-8-Deoxycumambrin B	*Pyrethrum pyrethroides* B. Fedtsch.	PMMA/CDCl_3_	2010	Relative configuration	[76]
(−)-Dibromopalau’amine	*Stylotella agminata*	PAN/DMSO	2010	Absolute configuration	[77]
Parthenolide	*Chrysanthemum parthenium*	PAN/DMSO	2010	Conformation	[78]
Fibrosterol sulfate A	*Lissodendoryx (Acanthodoryx)* fi*brosa*	dPAN/DMSO	2011	Relative configuration	[79]
Asperdimin dimer analog	*Asperdimin* alkaloid	PDMS/CDCl_3_	2011	Relative and absolute configuration	[80]
Vatiparol	*Vatica parvifolia*	PH/DMSO	2012	Absolute configuration	[81]
19-OH-(−)-eburnamonine	*Bonafusia macrocalyx*	PMMA/CDCl_3_	2012	Absolute configuration	[82]
4,6-Diacetylhygrophorone A^12^	*Hygrophorus*	PBLG/CD_2_Cl_2_	2013	Relative configuration	[83]
Rifamycin-S	*Amycolatopsis rifamycinica*	PDMS/CDCl_3_	2013	Relative configuration, conformation	[84]
Cyclolinopeptide A	*Linum*	PDMS/CDCl_3_	2014	Conformation	[85]
Melohenine B	*Melodinus henryi*	PBLG/CDCl_3_	2015	Conformation	[86]
α-Santonin	*Artemisia Santonica*	PAN/DMSO	2015	Relative configuration	[87]
Retrorsine	*Senecio vulgaris*	p-HEMA, PMMA/DMSO	2016	Conformation	[88]
Fusariumin A	*Fusarium* sp.	PH/DMSO	2016	Absolute configuration	[89]
Dihydroartemisin	*Artemisia*	GO-*g*-TFEMA/DMSO	2016	Absolute configuration	[24]
Homodimericin A	*Aspergillus niger*	p-HEMA/DMSO	2016	Relative configuration	[90]
β-Caryophyllene	*Syzygium aromaticum*	PDMS/CDCl_3_	2017	Conformation	[39]
Xylorumphiins	*Xylocarpus rumphii*	PMMA/CDCl_3_	2017	Relative configuration	[91]
Methylgriselimycin	*Streptomyces griseus*	PDMS/CDCl_3_	2017	Conformation	[92]
Cryptospirolepine	*Cryptolepis sanguinolenta*	p-HEMA/DMSO	2017	Relative configuration	[93]
Aquatolide	*Asteriscus aquaticus*	p-HEMA/DMSO	2017	Relative configuration	[93]
Ecteinamycin	*Actinomadura* sp.	PMMA/CDCl_3_	2017	Relative configuration	[94]
β-Heptapeptide	β-HXaa	PVAc/CD_3_OD	2017	Relative configuration	[95]
Gibberellin	*Gibberella fujikuroi*	AAKLVFF/CD_3_OD	2017	Absolute configuration	[22]
Ingenol	*Croton tiglium* L.	AAKLVFF/CD_3_OD	2017	Absolute configuration	[22]
Ginkgolide B	*Ginkgo biloba*	AAKLVFF/CD_3_OD	2017	Absolute configuration	[22]
Actinomycin D	*streptomyces* sp.	AAKLVFF/CD_3_OD	2017	Absolute configuration	[22]
Artemether	*Artemisia*	AAKLVFF/CD_3_OD	2017	Absolute configuration	[22]
Caulamidine A	*Calibugula intermis*	p-HEMA/CD_3_CN	2018	Absolute configuration	[96]

## Conclusion and Outlook

The application of RDCs for skeletal, configurational, and conformational analyses of organic small molecules in orienting media has been developed in the last decades and the field is ever growing. Following the conceptual introduction, this review has mentioned a selection of examples to illustrate the tour-de-force of RDCs in elucidating complex structures of natural products.

As a developing field, there is still a plenty of room for further improvements. The lack of commercially available alignment media is one of the most serious impediments before this technique can be adopted routinely. Therefore, the development of new alignment media, particularly new chiral media should be further pursued. Employing strategies of organic synthesis and polymer chemistry, new media should be rationally designed and tested to give deeper insights into the (enantiodiffentiating) alignment interactions. Furthermore, the efforts aimed at acquiring long-range RDCs will gain further applicability by the development of the new desired pulse sequences, the application of these new developments should not only improve precision and accuracy of heteronuclear RDCs but also allow access to the rich structure information content homonuclear RDCs. The broad applicability and success of computer-assisted 3D structure elucidation (CASE-3D) strategy based on the use of NMR parameters notwithstanding, new developments are needed to complement and improve upon this established method [97, 98]. The new program such as MSpin and MSpin-JCoupling etc. [99] should be revisited or developed to help organic and bioorganic chemists in the structural elucidation of organic compounds through use of RDCs.

RDCs will keep contributing to a variety of structure determination problems and will become an important tool for all NMR spectroscopists, or in combination with the measurement of residual chemical shift anisotropies (RCSAs) [100] and deuterium residual quadrupolar couplings (^2^H-RQCs) [101] in weakly oriented (chiral) solvents. With the development of anisotropic NMR parameters, in the near future, this methodology will be routinely applicable for assigning the stereochemical correlation of spatially distant stereocenters in structurally complicated molecules.
